# Editorial: Novel pharmacological treatments in sleep disorders

**DOI:** 10.3389/fnins.2022.1086983

**Published:** 2022-12-01

**Authors:** Roberto De Luca, William D. Todd, Christian R. Burgess

**Affiliations:** ^1^Division of Sleep Medicine, Department of Neurology, Beth Israel Deaconess Medical Center, Harvard Medical School, Boston, MA, United States; ^2^Program in Neuroscience, Department of Zoology and Physiology, University of Wyoming, Laramie, WY, United States; ^3^Department of Molecular and Integrative Physiology and Michigan Neuroscience Institute, University of Michigan, Ann Arbor, MI, United States

**Keywords:** sleep, inflammation, neuroinflammation, circadian rhythm disorders, melanin concentrating hormone (MCH)

This collection offers an insightful overview about some recent advancements in identifying neuronal targets, circuits, and potential pharmacological treatments to combat sleep-related disorders. Poor sleep increases the risk of developing a wide range of disorders, from neurological to metabolic, that compromise the health of the effected individuals. Developing effective and specific treatments for sleep disorders represent a real challenge, mainly due to the difficulty in finding drugs that show a selective action on defined targets without causing unwanted side effects. Therefore, controlling the symptoms of the sleep-related disorders, with highly selective drugs, is an important goal for this field. In this issue of Frontiers in Neuroscience—section: Sleep and Circadian Rhythms - the contributing authors described novel circuits and mechanisms involved in sleep disorders. Our special issue comprises articles that aim to fill the gaps in our understanding of the regulation of sleep, addressing both well-known and novel mechanisms through which new targets for pharmacological intervention could be identified. A particular focus regards the intriguing link between sleep deprivation (SD), inflammation and neuro-inflammation (Figure 1).

**Figure 1:**
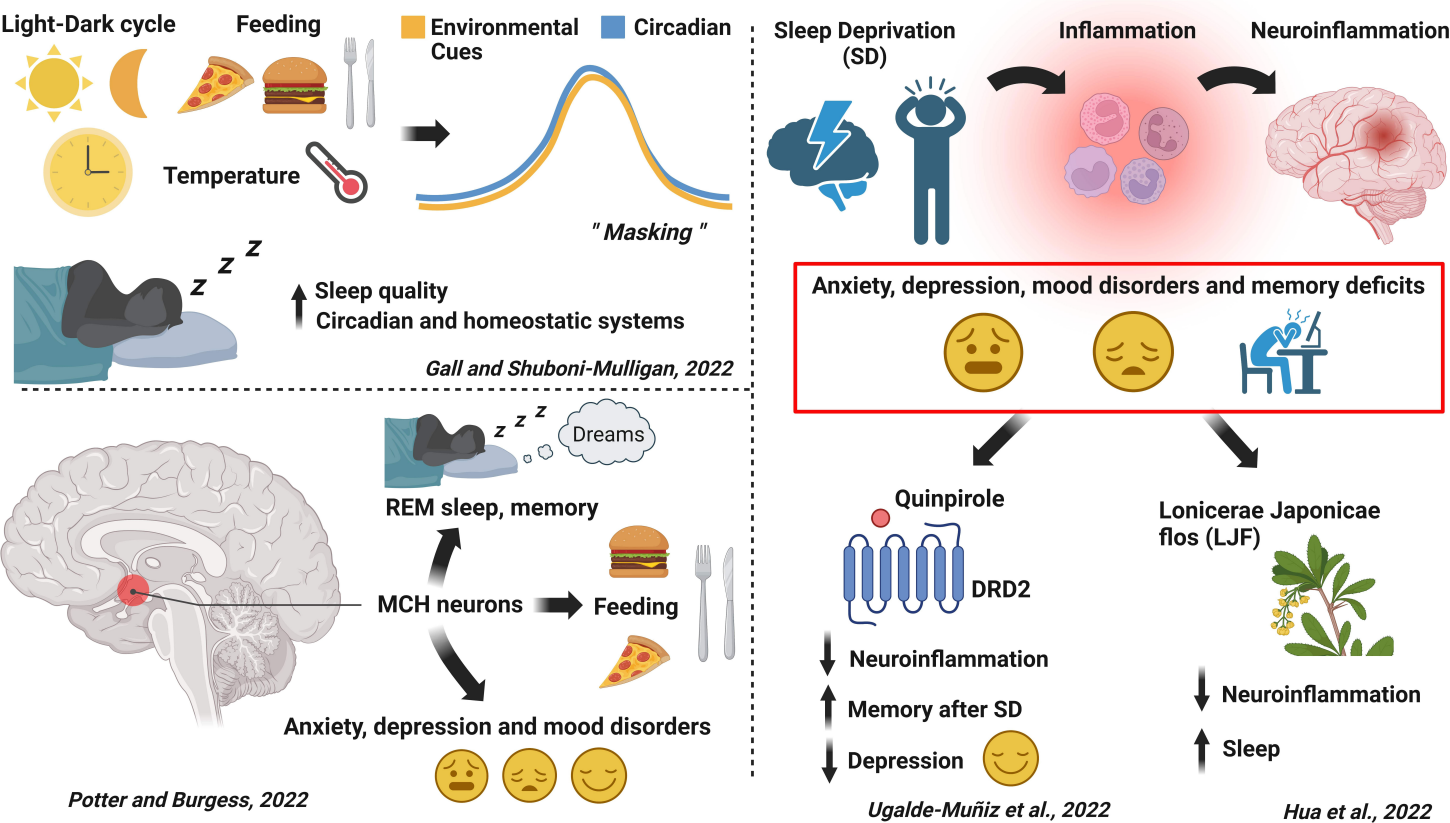
A schematic representation describing the major findings that the contributing articles provided to our special issue and that highlight the novel targets and potential pharmacological treatments to treat sleep and circadian disorders (created with BioRender.com).

The review by Gall and Shuboni-Mulligan looks at the phenomenon of masking, arguing that masking is a separate factor which contributes to the patterns of behavior, not simply as something that obscures endogenous circadian rhythms. The authors review evidence of various exogenous stimuli that can act as masking agents, demonstrating their ability to interact with homeostatic and circadian systems, and discuss the neural circuitry involved. There is the potential for masking agents to be used therapeutically to improve abnormal and maladaptive sleep behaviors resulting from primary sleep disorders and/or sleep disruptions that are secondary to other illnesses. Using masking stimuli themselves, or pharmacological approaches targeting the neural circuitry through which masking occurs could be a valuable approach in treating disordered sleep. In a similar vein, the review by Potter and Burgess explores the history and promise of the melanin concentrating hormone (MCH) system as a target for treating sleep disorders. MCH containing neurons project throughout the brain and have been implicated in a number of important physiological processes, including regulation of REM sleep. This makes it a potentially attractive target for drugs aimed at disorders of REM sleep, such as narcolepsy. As the review details, MCH also affects consummatory behavior and there were several attempts to develop MCH-based drugs or derivatives for obesity, but these have proven to be difficult to bring to market. However, given the extent of basic science demonstrating the power of MCH to influence sleep, this remains a viable target for development of new tools to treat sleep disorders.

It has been extensively demonstrated that getting low-quality or little sleep can be detrimental for memory performance and, at the same time, it can be a significant factor for the development of anxiety and depression-related behaviors. Interestingly, it has also been reported that SD causes alterations of immune function and that neuroinflammation, as consequence of sleep disturbances, could play an important role in worsening cognition or in inducing rapid mood changes. In this respect, the work published by Ugalde-Muñiz et al. described the neuroprotective role of dopamine (DA) and the activation of DA receptor 2 (DRD2) that is involved in the reduction of inflammation caused by SD. In mice, REM sleep deprivation (RSD) increased proinflammatory cytokines (TNFα and IL-1β) in the hippocampus and serum and was sufficient to induce inflammation. In general, SD causes memory impairments and immunological alterations by increasing the levels of TNFα, IL-1β, and IL-6. In this study, an emerging role of DA in modulating the immune response was described. Agonism at the level of DRD2 by quinpirole reduced inflammation caused by RSD and also recovered spatial memory impairments in RSD mice. At the same time, RSD mice showed inflammation within the hippocampus suggesting that DRD2 could be a potential target for treating memory deficits induced by RSD. However, DRD2 activation *per se* does not improve the mnemonic capabilities. Quinpirole also prevented a depressive-like behavior caused by RSD. This neuropathological status, as well as the anhedonia behavior observed in RSD mice, is indeed characterized by high levels of IL-1β, IL-6, and TNFα, as a consequence of the inflammatory state triggered by SD.

Finally, a new chemical compound was tested to potentially treat inflammation caused by sleep disturbances. In the work of Hua et al., the effect of Lonicerae Japonicae flos (LJF) was explored. The LJF extracts are able to reduce and downregulate the production of inflammatory cytokines. Among the components of LJF extracts, chlorogenic acid and luteolin are linked to the increase of sleep time and reduction of sleep latency. Very little is known about the mechanisms by which LJF can regulate sleep homeostasis. The aim of this study was to understand the action of LJF on sleep-wake cycle under normal conditions, in SD, and with lipopolysaccharide (LPS) treatment, which induces inflammation in animals. As previously discussed in the work of Ugalde-Muñiz et al., SD is associated with the increase of inflammatory cytokines such as IL-6 and TNFα. Under basal conditions, LJF increases the time spent in non-REM sleep and reduced wakefulness. Moreover, LJF helped sleep recovery after acute SD. In another set of experiments, the authors found that LJF promoted REM sleep after injections of LPS in mice. Another effect observed was the capability of LJF to decrease the levels of proinflammatory cytokines in both blood serum and brain tissues in mice treated with LPS. The authors concluded that LJF inhibits microglial activation in hippocampus and in medial prefrontal cortex to reduce the inflammation. This study highlights the potential of LJF, as a new compound, to be used in clinics for the treatment of neuroinflammatory conditions linked to SD.

## Conclusions

This collection of articles explores new avenues of research directed at some key neuronal and cellular players that could be critical pharmacological targets to treat sleep and circadian disorders. Moreover, a novel focus on the crosstalk between neuronal and immune systems has been described and may represent one of the most promising directions to consider in future.

## Author contributions

All authors listed have made a substantial, direct, intellectual and equal contribution to the work, and approved it for publication.

## Funding

This work was funded by NIH 1R01DK129366-01 to CB and 2P20GM121310-06 from NIGMS to WT.

## Conflict of interest

The authors declare that the research was conducted in the absence of any commercial or financial relationships that could be construed as a potential conflict of interest.

## Publisher's note

All claims expressed in this article are solely those of the authors and do not necessarily represent those of their affiliated organizations, or those of the publisher, the editors and the reviewers. Any product that may be evaluated in this article, or claim that may be made by its manufacturer, is not guaranteed or endorsed by the publisher.

